# EngineeRING the gear of ATP synthase: a way to address complexity and flexibility of photosynthesis

**DOI:** 10.1093/plphys/kiad148

**Published:** 2023-03-06

**Authors:** Ryo Yokoyama

**Affiliations:** Assistant Features Editor, Plant Physiology, American Society of Plant Biologists, USA; Max-Planck-Institute of Molecular Plant Physiology, Am Mühlenberg 1, Potsdam-Golm, 14476, Germany

ATP synthase plays a crucial role in ATP production in all organisms. The F_0_ ring region of ATP synthase is rotated by protons translocated across the membranes in response to proton motive force (pmf)—a transmembrane energy source composed of the membrane potential (Δψ) and the proton concentration gradient (ΔpH) (Davis et al. 2017).

Despite their conserved role and mechanism in ATP production, there is structural diversity in the number of transmembrane proteins, called c-subunits, that form the ring rotor. The c-ring stoichiometry differs among bacteria, animals, and photosynthetic organisms, from c_8_ in animal mitochondria (Watt et al. 2010) and c_14_ in plant chloroplasts (Seelert et al. 2000; Hahn et al. 2018) to c_15_ in the cyanobacteria *Burkholderia platensis* (Pogoryelov et al. 2005). The c-ring stoichiometry determines how many protons are required to generate one ATP molecule. An ATP synthase with a larger c-ring stoichiometry needs more protons per ATP synthesized, making ATP synthase less energy-efficient (Nesci et al. 2016; Cheuk and Meier 2021).

Why do chloroplasts rely on an ATP synthase with a higher c-ring number that carries a cost in proton flux? As Davis and Kramer (2020) described, the c-ring acts like a bicycle gear to optimize its efficiency with power needs depending on the road conditions. This analogy makes it easier to imagine that the c-subunit stoichiometry in the chloroplast ATP synthase is fixed as a result of the adjustment to local photosynthetic conditions in which multiple factors are involved. For example, the characterization of Arabidopsis (*Arabidopsis thaliana*) loss-of-function and gain-of-function mutants of the thylakoid-localized K^+^ exchange antiporter 3 (KEA3) reveals that KEA3 mediates proton export from the lumen side, making the Δψ/ΔpH ratio higher with little impact on total pmf size (Armbruster et al. 2014; Wang et al. 2017). However, comprehensive understanding of the regulatory mechanism of the pmf formation/relaxation remains challenging.

Yamamoto et al. (2023) tackled this question by engineering the c-subunit stoichiometry of tobacco (*Nicotiana tabacum*) ATP synthase. With the idea that changing the ring size can be utilized to analyze the impact of the engineered ATP synthase on photosynthesis, the authors expressed the cyanobacterial gene encoding the c_15_-ring subunit in the background of the knockout albino mutant of the *atpH* gene (Fig. 1), which encodes the c-subunit in *N. tabacum*. To their surprise, the engineered ATP synthase was functional enough for the successful transformants to grow well, despite the decreased ATPase complex abundance to only 25% of the wild-type level. One previous study suggested that a similar reduction in ATP synthase levels severely impaired growth (Rott et al. 2011). The enhanced proton conductivity and proton flux through the engineered ATP synthase were monitored in the c_15_ transgenic lines (Fig. 1). This result demonstrated that the pmf, the driving force behind ATP synthase, was consumed more rapidly in the transgenic lines, accelerating the pmf formation primarily due to an increased Δψ without affecting photosynthetic electron transport. Since this phenotype was similar to what was observed in an Arabidopsis ion channel mutant (Duan et al. 2016), it appears that an optimized ion permeability across thylakoid membranes might elevate pmf in the transgenic plants, contributing to a higher proton flux through ATP synthase.

**Figure 1.**
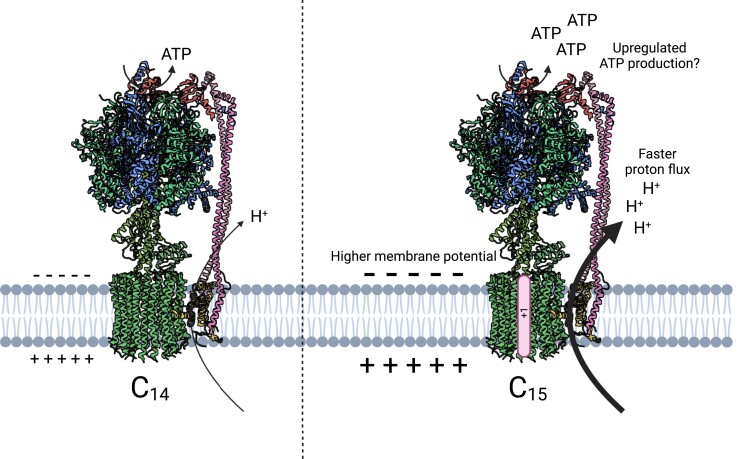
The model of ATP synthase with different c-ring stoichiometry. The model structures of wild-type ATP synthase having the c_14_ ring (left) and engineered ATP synthase having the c_15_ ring from the cyanobacteria *Burkholderia platensis* (right). Spinach (*Spinacia oleracea*) chloroplast ATP synthase structure (PDB 6FKF) was mapped to this figure (Hahn et al. 2018).

What lessons does the engineered ATP synthase rotor ring provide us? Readjustment of the pmf profiling by artificial remodeling of ATP synthase functionality highlights the dynamic flexibility and complexity of photosynthesis. This finding might not be addressed by simple knockout/down or overexpression experiments. In theory, the increased c-ring stoichiometry results in a decrease in the ATP synthase efficiency (Cheuk and Meier 2021). Unexpectedly, however, the transformants expressing the enlarged ATP synthase ring grew well with faster proton flux through ATP synthase. One possible scenario is that the modified ATP synthase, with the enlarged c-ring, is more actively driven in response to a higher level of pmf that might be generated by altered regulation of the ion channel/transporter activities. As a result, enhanced ATP production might be sufficient to maintain plant growth even in a situation where the abundance of ATP synthase was drastically reduced. However, direct evidence of elevated ATP generation in vivo is lacking in this report and will be needed to test the hypothesis in future studies. Note that in vivo measurement of dynamic ATP production kinetics in chloroplasts is technically challenging.

A list of the next tasks includes elucidating how ion permeability across thylakoid membranes flexibly responds to adjust the pmf component for maintaining plant growth and whether other factors, such as cyclic electron flow around Photosystem I (Wang et al. 2015), are involved in this step, which will help us understand the mechanism behind the photosynthetic adaptation in wild-type plants to harsh light conditions. A possible answer to these questions may provide insight into why the number of ATP synthase ring subunits in plant chloroplasts is conserved at 14.

## References

[kiad148-B1] Armbruster U , Carrillo LR, Venema K, Pavlovic L, Schmidtmann E, Kornfeld A, Jahns P, Berry JA, Kramer DM, Jonikas MC. Ion antiport accelerates photosynthetic acclimation in fluctuating light environments. Nat Commun. 2014:5(1): 5439. 10.1038/ncomms6439 25451040 PMC4243252

[kiad148-B2] Cheuk A , Meier T. Rotor subunits adaptations in ATP synthases from photosynthetic organisms. Biochem Soc Trans. 2021:49(2): 541–550. 10.1042/BST20190936 33890627 PMC8106487

[kiad148-B3] Davis GA , Kramer DM. Optimization of ATP synthase c–rings for oxygenic photosynthesis. Front Plant Sci. 2020:10: 1778. 10.3389/fpls.2019.01778 32082344 PMC7003800

[kiad148-B4] Davis GA , Rutherford AW, Kramer DM. Hacking the thylakoid proton motive force for improved photosynthesis: modulating ion flux rates that control proton motive force partitioning into Δψ and ΔpH. Philos Trans R Soc Lond B Biol Sci. 2017:372(1730): 20160381. 10.1098/rstb.2016.0381 28808100 PMC5566881

[kiad148-B5] Duan Z , Kong F, Zhang L, Li W, Zhang J, Peng L. A bestrophin-like protein modulates the proton motive force across the thylakoid membrane in Arabidopsis. J Integr Plant Biol. 2016:58(10): 848–858. 10.1111/jipb.12475 26947269 PMC5074266

[kiad148-B6] Hahn A , Vonck J, Mills DJ, Meier T, Kühlbrandt W. Structure, mechanism, and regulation of the chloroplast ATP synthase. Science. 2018:360(6389): eaat4318. 10.1126/science.aat4318 29748256 PMC7116070

[kiad148-B7] Nesci S , Trombetti F, Ventrella V, Pagliarani A. The c-ring of the F1FO-ATP synthase: facts and perspectives. J Membr Biol. 2016:249(1–2): 11–21. 10.1007/s00232-015-9860-3 26621635

[kiad148-B8] Pogoryelov D , Yu J, Meier T, Vonck J, Dimroth P, Muller DJ. The c_15_ ring of the *Spirulina platensis* F-ATP synthase: F_1_/F_0_ symmetry mismatch is not obligatory. EMBO Rep. 2005:6(11): 1040–1044. 10.1038/sj.embor.7400517 16170308 PMC1371026

[kiad148-B9] Rott M , Martins NF, Thiele W, Lein W, Bock R, Kramer DM, Schöttler MA. ATP synthase repression in tobacco restricts photosynthetic electron transport, CO_2_ assimilation, and plant growth by overacidification of the thylakoid lumen. Plant Cell. 2011:23(1): 304–321. 10.1105/tpc.110.079111 21278125 PMC3051256

[kiad148-B10] Seelert H , Poetsch A, Dencher NA, Engel A, Stahlberg H, Müller DJ. Structural biology. Proton-powered turbine of a plant motor. Nature. 2000:405(6785): 418–419. 10.1038/35013148 10839529

[kiad148-B11] Wang C , Yamamoto H, Narumiya F, Munekage YN, Finazzi G, Szabo I, Shikanai T. Fine-tuned regulation of the K^+^/H^+^ antiporter KEA3 is required to optimize photosynthesis during induction. Plant J. 2017:89(3): 540–553. 10.1111/tpj.13405 27783435

[kiad148-B12] Wang C , Yamamoto H, Shikanai T. Role of cyclic electron transport around photosystem I in regulating proton motive force. Biochim Biophys Acta. 2015:1847(9): 931–938. 10.1016/j.bbabio.2014.11.013 25481109

[kiad148-B13] Watt IN , Montgomery MG, Runswick MJ, Leslie AGW, Walker JE. Bioenergetic cost of making an adenosine triphosphate molecule in animal mitochondria. Proc Natl Acad Sci U S A. 2010:107(39): 16823–16827. 10.1073/pnas.1011099107 20847295 PMC2947889

[kiad148-B14] Yamamoto H , Cheuk A, Shearman J, Nixon PJ, Meier T, Shikanai T. Impact of engineering the ATP synthase rotor ring on photosynthesis in tobacco chloroplasts. Plant Physiol. 2023:192(2):1221–1233. 10.1093/plphys/kiad043 PMC10231360 36703219

